# Proinflammatory Effects of IL-1β Combined with IL-17A Promoted Cartilage Degradation and Suppressed Genes Associated with Cartilage Matrix Synthesis In Vitro

**DOI:** 10.3390/molecules24203682

**Published:** 2019-10-13

**Authors:** Patiwat Kongdang, Chatchadawalai Chokchaitaweesuk, Siriwan Tangyuenyong, Siriwan Ongchai

**Affiliations:** 1 Department of Biochemistry, Faculty of Medicine, Chiang Mai University, 110 Inthawarorot Road, Chiang Mai 50200, Thailand; patiwat.kongdang@gmail.com (P.K.); chatchada1003@gmail.com (C.C.); 2 Graduate School, Chiang Mai University, 239 Huay Kaew Road, Chiang Mai 50200, Thailand; 3 Equine Clinic, Department of Companion Animal and Wildlife Clinic, Faculty of Veterinary Medicine, Chiang Mai University, Chiang Mai 50100, Thailand; makhaboocha@gmail.com; 4 Thailand Excellence Center for Tissue Engineering and Stem Cells, Department of Biochemistry, Faculty of Medicine, Chiang Mai University, 110 Inthawarorot Road, Chiang Mai 50200, Thailand

**Keywords:** cartilage-specific anabolic gene, cytokine combination, inflammatory arthritis, porcine cartilage explant model, porcine pellet culture model, proinflammatory cytokine

## Abstract

Combinations of IL-1β and other proinflammatory cytokines reportedly promote the severity of arthritis. We aimed to investigate the effects of IL-1β combined with IL-17A on cartilage degradation and synthesis in in vitro models. Cartilage explant degradation was determined using sulfated glycosaminoglycans (S-GAGs) levels, matrix metalloproteinase (MMP13) gene expression, uronic acid, and collagen contents. Cell morphology and accumulation of proteoglycans were evaluated using hematoxylin-eosin and safranin O staining, respectively. In the pellet culture model, expressions of cartilage-specific anabolic and catabolic genes were evaluated using real-time qRT-PCR. Early induction of MMP13 gene expression was found concomitantly with significant S-GAGs release. During the prolonged period, S-GAGs release was significantly elevated, while MMP-13 enzyme levels were persistently increased together with the reduction of the cartilaginous matrix molecules. The pellet culture showed anabolic gene downregulation, while expression of the proinflammatory cytokines, mediators, and MMP13 genes were elevated. After cytokine removal, these effects were restored to nearly basal levels. This study provides evidence that IL-1β combined with IL-17A promoted chronic inflammatory arthritis by activating the catabolic processes accompanied with the suppression of cartilage anabolism. These suggest that further applications, which suppress inflammatory enhancers, especially IL-17A, should be considered as a target for arthritis research and therapy.

## 1. Introduction

Proinflammatory cytokines are critical factors involved in the pathogenesis of inflammatory arthritis, which triggers, amplifies, and prolongs the catabolic progression of joint diseases [1]. A key inflammatory cytokine, interleukin (IL)-1β, induces an acute inflammation such as osteoarthritis (OA) and extends the inflammatory loop together with other proinflammatory cytokines and inflammatory mediators such as IL-6, tumor necrosis factor alpha (TNF-α), cyclooxygenase-2 (COX2), and prostaglandin E2 (PGE2) [2]. IL-1β induces the production of cartilage-degrading enzymes, including matrix metalloproteinases (MMPs) and inhibits the synthesis of the cartilaginous extracellular matrix (ECM) in the chondrocytes [2]. IL-17A or IL-17 is involved in several chronic diseases, including rheumatoid arthritis (RA), and it upregulates other proinflammatory cytokines, including IL-1β and IL-6 [3]. IL-17-producing cells, that is, Th17 cells, are activated by IL-1β to produce IL-17, which also induces the production of other proinflammatory cytokines, including IL-1β in the activation loop [4]. In the animal joint system, IL-17A induces the mRNA expression of MMPs and suppresses COL2A1 gene expression in the cartilage [5].

MMP-13 is a key inducible catabolic factor in arthritis, and it efficiently cleaves type II collagen and other ECM molecules in articular cartilage, leading to the rapid loss of cartilaginous matrix [6]. Inflammatory cytokines induce the gene expression of MMPs in the cartilage; however, these are regulated by the tissue inhibitors of metalloproteinases (TIMPs) as an endogenous tissue inhibitor [7]. Therefore, the ratio of MMP13/TIMP1 gene expression is used as a marker for cartilage degradation [8].

Various types of inflamm atory cytokines act together in the progression of joint inflammation. IL-17A was noted to synergistically contribute to multiple proinflammatory cytokines such as IL-1, IL-6, oncostatin M (OSM), and TNF-α for the induction of inflammatory arthritis, including synovial hyperplasia, cartilage destruction, and ECM breakdown [9,10,11]. Recently, a treatment comprising IL-1β in combination with IL-17A reportedly enhanced the expression of genes associated with arthritis and cell migration in a monolayer culture of the human synovial sarcoma cell line [12].

Since patients with arthritis have a heterogeneous condition, the effects of single cytokine-targeting therapies were noted vary followed by a poor prognosis [13]. Targeting a combination of proinflammatory cytokines may have clinical efficacy in anti-inflammatory arthritis. However, there is inadequate evidence of the effect of the cytokine combination on the cartilaginous matrix biosynthesis. Despite the anabolic recovery, the recurrence of inflammatory arthritis after the withdrawal of the combined cytokine treatment remains unknown.

Therefore, we aimed to investigate the effects of the combination of IL-1β and IL-17A treatment on cartilage degradation and matrix synthesis using a porcine cartilage explant culture model. The porcine pellet culture model was adopted for investigating the effects of the cytokine combination on the expression levels of cartilage-specific anabolic genes along with the catabolic genes. The ability to restore the matrix regenerating ability of the affected chondrocytes was also investigated.

## 2. Results

### 2.1. Cartilage Explant Culture Model

**Figure 1:**
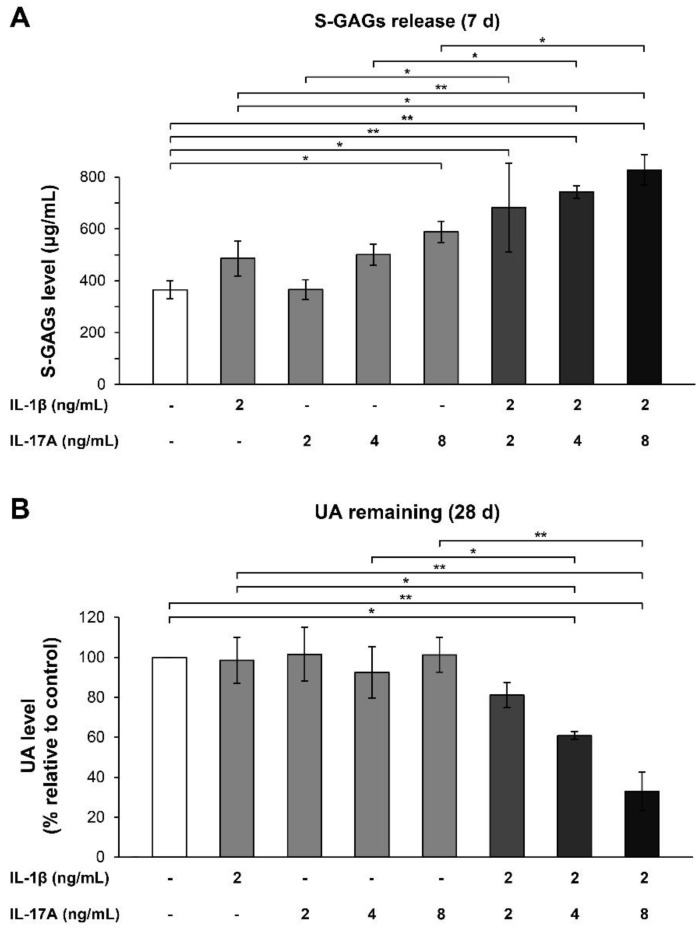
Combinatorial effects of IL-1β and IL-17A on the levels of S-GAGs release and UA content in porcine cartilage explant culture. The explants were cultured for 7 and 28 days under conditions, which stimulated by IL-1β, IL-17A, and the combination of these cytokines at indicated concentrations. Levels of S-GAGs release were measured in culture media (**A**), and papain-digested cartilages were determined for UA content (**B**). Data are expressed as mean ± SD of three independent experiments. * = *p* < 0.05 and ** = *p* < 0.001.

The porcine cartilage explants were stimulated with either IL-1β or IL-17A alone or IL-1β in combination with different doses of IL-17A for optimization of the cartilage-degrading system. IL-17A enhanced the effects of IL-1β by increasing the release of sulfated glycosaminoglycans (S-GAGs) into the culture media concomitantly with a great reduction in the uronic acid (UA) content in the cartilage tissues (Figure 1). Significant changes in S-GAGs release and the remaining UA content were observed when 2 ng/mL of IL-1β was used in combination with IL-17A at concentrations of 4–8 ng/mL. Therefore, this combination of cytokines was used for treatment in the cartilage explant model for both short-term and long-term treatments.

#### 2.1.1. Short-Term Cartilage Explant Culture Model

**Figure 2:**
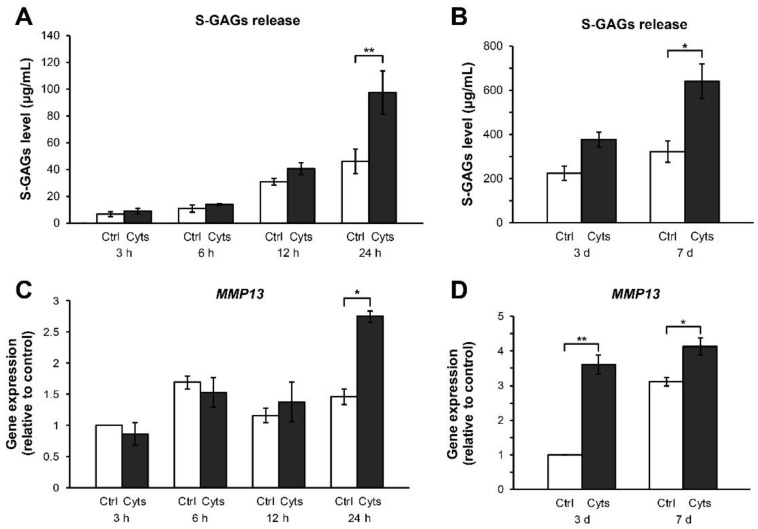
Effects of the IL-1β and IL-17A combination on the levels of S-GAGs release and MMP13 gene expression in short-term porcine cartilage explant culture. The explants were cultured for 1, 3, and 7 days under conditions in which they were stimulated by the combined cytokines (Cyts; 2 ng/mL of IL-1β in combination with 4 ng/mL of IL-17A). The untreated explants were left as a control (Ctrl). Levels of S-GAGs release in culture media (**A**,**B**) and MMP13 gene expression (**C**,**D**) were determined at indicated times. Data are expressed as mean ± SD of three independent experiments. * = *p* < 0.05 and ** = *p* < 0.001.

Using a combination treatment of IL-1β and IL-17A for 24 h and up to 7 days, we investigated the cartilage degradation to reveal the effects of combined cytokine exposure on the early response of the cartilage explants. The levels of S-GAGs released in the culture media were significantly increased at 24 h after treatment compared with those in the untreated control (Figure 2A). Cartilage destruction was evaluated for a week. Combined cytokine treatments continuously increased the levels of S-GAGs release from 3–7 days (Figure 2B). The gene expression of MMP13 was highly induced by the combined cytokine treatment at 24 h (Figure 2C) and reached a maximum level at 3–7 days (Figure 2D).

#### 2.1.2. Long-Term Cartilage Explant Culture Model

**Figure 3:**
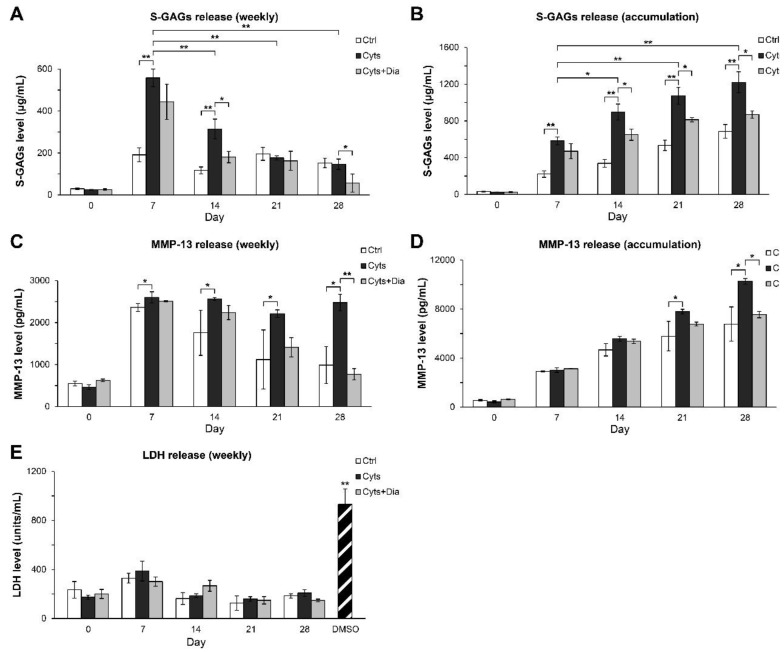
Effects of the combination of IL-1β and IL-17A on the release of S-GAGs and MMP-13 in long-term porcine cartilage explant culture. The explants were cultured for 28 days under conditions in which they were stimulated by the combined cytokines (Cyts; 2 ng/mL of IL-1β in combination with 4 ng/mL of IL-17A) in with or without diacerein (Dia; 50 µM). The untreated explants were left as a control (Ctrl). Levels of S-GAGs release (**A**,**B**), MMP-13 protein (**C**,**D**), and LDH release (**E**) in culture media were determined weekly. Data are expressed as mean ± SD of three independent experiments. * = *p* < 0.05 and ** = *p* < 0.001.

The long-term treatment of cartilage explants was performed for 28 days. Figure 3A,B illustrates the weekly levels of S-GAGs released into the culture media and its accumulation pattern. The combination treatment with IL-1β and IL-17A drastically stimulated S-GAGs release with the maximum increase in the levels of S-GAG release observed at 7 days, after which these levels gradually decreased. Diacerein, a slow-acting drug for joint diseases, significantly reduced the levels of S-GAGs release at 14 and 28 days. The accumulation levels of S-GAGs increased upon combined cytokine treatment for up to 28 days, whereas diacerein attenuated this increase. The MMP-13 protein was continuously released in significant amounts from 7 days, and diacerein strongly suppressed this release from 21 days, reaching baseline levels at 28 days (Figure 3C,D). In the long-term culture, the levels of LDH release in all the treatment groups were similar those in the untreated control group compared with the substantial levels of LDH release observed for the DMSO positive control treatment (Figure 3E).

**Figure 4:**
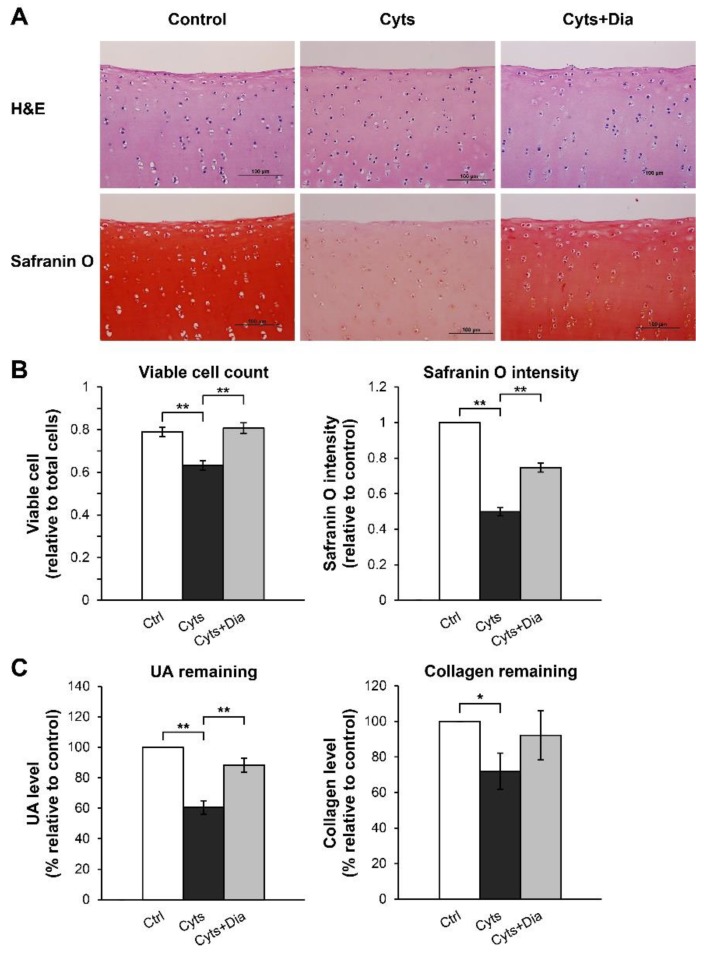
Effects of the IL-1β and IL-17A combination on the remaining chondrocytes and matrix molecules in long-term porcine cartilage explant culture. The explants were cultured for 28 days under conditions in which they were stimulated by the combined cytokines (Cyts; 2 ng/mL of IL-1β in combination with 4 ng/mL of IL-17A) with or without diacerein (Dia; 50 µM). The untreated explants were left as a control (Ctrl). The cartilages were stained with H&E and safranin O dyes for an observation of chondrocytes and matrix molecules (**A**). Viable cells were counted on H&E-stained slides, and the intensities of safranin O were calculated on safranin O-stained slides (**B**). Papain-digested cartilages were analyzed using the remaining of UA and collagen (**C**). Data are expressed as mean ± SD of three independent experiments. * = *p* < 0.05 and ** = *p* < 0.001.

The histopathological examination of H&E-stained sections at 28 days showed a normal rounded structure of the chondrocytes embedded in the cartilage tissue (Figure 4A). The chondrocyte morphology in each group did not differ from that in the untreated control group. However, the number of viable chondrocytes with nucleated lacunae was reduced in the combined cytokine group, whereas a similar number of viable cells were observed in the cotreated combined cytokines and diacerein group and the untreated control group (Figure 4B). Proteoglycans accumulation was significantly higher in the cotreated combined cytokine and diacerein group than in the combined cytokine treatment group (without the addition of diacerein), as evaluated by safranin O staining. Similar to the remaining cartilaginous ECM, UA and collagen contents in the cartilage explants were significantly lower in the combined cytokine treatment group than in the untreated control group. Cotreated combined cytokines and diacerein induced an increase in UA and collagen contents by 28% and 20%, respectively, compared with that in the combined cytokine treatment without diacerein (Figure 4C).

### 2.2. Pellet Culture Model

#### 2.2.1. Time-Course Effect of the Combined Cytokines

**Figure 5:**
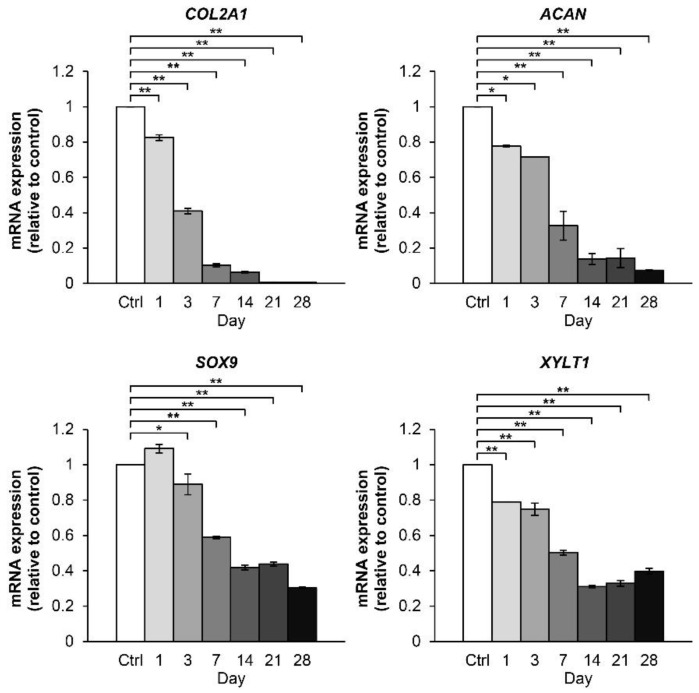
Time-course effect of the IL-1β and IL-17A combination on the expression of cartilaginous matrix anabolic genes in porcine pellet culture. The pellets were cultured up to 28 days under conditions in which they were stimulated by the combined cytokines (0.25 ng/mL of IL-1β in combination with 0.5 ng/mL of IL-17A). The untreated pellets were left as a control (Ctrl). The mRNA expressions of the pellets were analyzed by real-time qRT-PCR at the indicated times. Data are expressed as mean ± SD of two independent experiments. * = *p* < 0.05 and ** = *p* < 0.001.

The effects of the combination treatment with IL-1β and IL-17A on the expression of genes associated with cartilage metabolism were evaluated using the porcine articular chondrocyte pellet culture model. Two-fold serial dilutions of the combined cytokines, 2 ng/mL of IL-1β in combination with 4 ng/mL of IL-17A, were optimized for analyzing the mRNA expression levels of proinflammatory cytokines (Figure S4), inflammatory mediators (Figure S5), and cartilage-degrading enzyme (Figure S6). The concentrations of IL-1β and IL-17A selected for inflammatory stimulation in the porcine chondrocyte pellet culture model were 0.25 and 0.5 ng/mL, respectively. Cartilage-specific anabolic genes were significantly downregulated by the combined cytokine treatment in a time-dependent manner (Figure 5). The gene expression of COL2A1 was reduced to approximately half within 3 days, whereas a similar reduction in the gene expression levels of ACAN, SOX9, and XYLT1 was delayed by a week in this culture system. During long-term treatment with the combined cytokines, the gene expression of COL2A1 and ACAN were almost completely suppressed, whereas the SOX9 and XYLT1 genes showed basal expression levels until 28 days.

**Figure 6:**
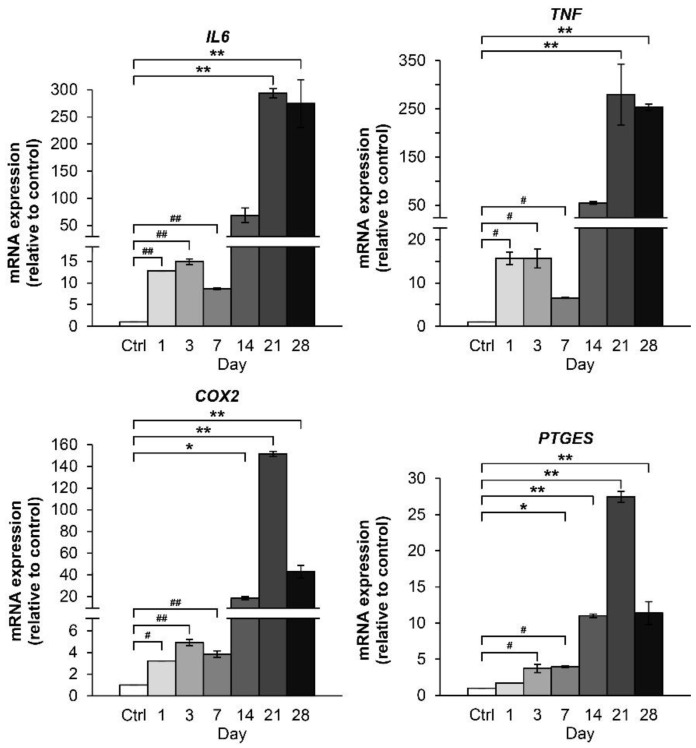
Time-course effect of the IL-1β and IL-17A combination on the mRNA expression of proinflammatory cytokines and inflammatory mediators in porcine pellet culture. The pellets were cultured up to 28 days under conditions in which they were stimulated by the combined cytokines (0.25 ng/mL of IL-1β in the combination with 0.5 ng/mL of IL-17A). The untreated pellets were left as a control (Ctrl). The mRNA expressions of the pellets were analyzed by real-time qRT-PCR at the indicated times. The symbol # indicates a statistical analysis within 7 days, and * indicates a statistical analysis within 28 days. Data are expressed as mean ± SD of two independent experiments. #, * = *p* < 0.05 and ##, ** = *p* < 0.001.

The expression levels of IL6 and TNF genes were highly upregulated by the combined cytokine treatment at 3 days, followed by a small decrease on 7 days (Figure 6). Beyond this reverse point at 7 days, the gene expression levels increased significantly from 14 days to 28 days. Conversely, the gene expression levels of COX2 and PTGES showed a gradual increase up to 21 days, at which point expression levels declined to half of the highest measured amount by 28 days.

**Figure 7:**
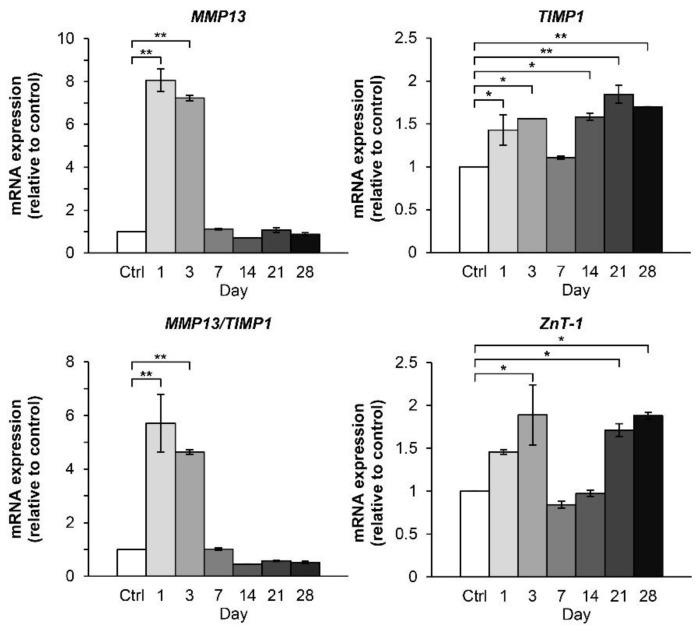
Time-course effect of the IL-1β and IL-17A combination on the expression of the genes associated with the cartilaginous matrix-degrading enzymes in porcine pellet culture. The pellets were cultured for up to 28 days under conditions in which they were stimulated by the combined cytokines (0.25 ng/mL of IL-1β in combination with 0.5 ng/mL of IL-17A). The untreated pellets were left as a control (Ctrl). The mRNA expressions of the pellets were analyzed by real-time qRT-PCR at the indicated times. Data are expressed as mean ± SD of two independent experiments. * = *p* < 0.05 and ** = *p* < 0.001.

The combined cytokine treatment significantly upregulated the gene expression of MMP13 and the ratio of MMP13: TIMP1 from 1 day to 3 days. However, these levels dropped at 7 days to level similar to those observed in the untreated control group (Figure 7). The expression levels of TIMP1, the MMP inhibitor, and ZnT-1, zinc transporter, in the combined cytokine treatment group showed a gradual slow-increase pattern in 28 days, after the turning point at 7 days.

#### 2.2.2. Withdrawal Effect of the Combined Cytokines

**Figure 8:**
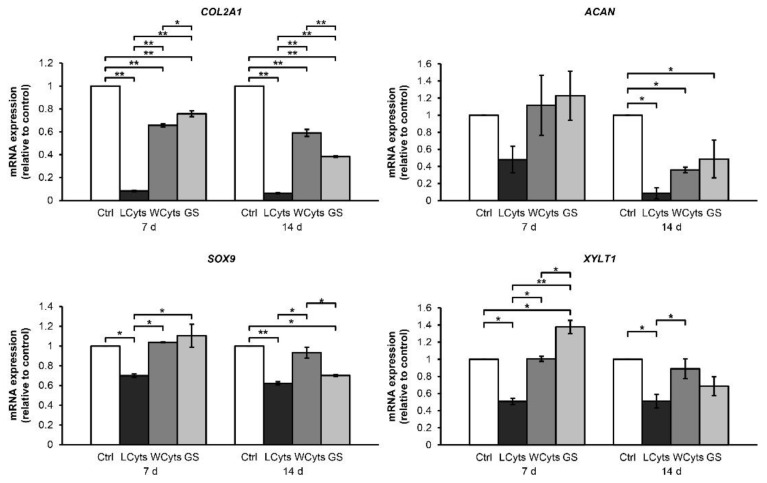
The cytokine-withdrawal effects after pretreatment with the IL-1β and IL-17A combination on the expression of cartilaginous matrix anabolic genes in porcine pellet culture. The pellets were pretreated by the combined cytokines (0.25 ng/mL of IL-1β in combination with 0.5 ng/mL of IL-17A) for 3 days, and then cultured for 7 and 14 days by non-cytokines treatments (Wcyts) with or without glucosamine sulfate (GS; 125 μg/mL). The untreated pellets were left as a control (Ctrl). Long-term treatments were continuously cultured until 7 and 14 days (LCyts). Data are expressed as mean ± SD of two independent experiments. * = *p* < 0.05 and ** = *p* < 0.001.

**Figure 9:**
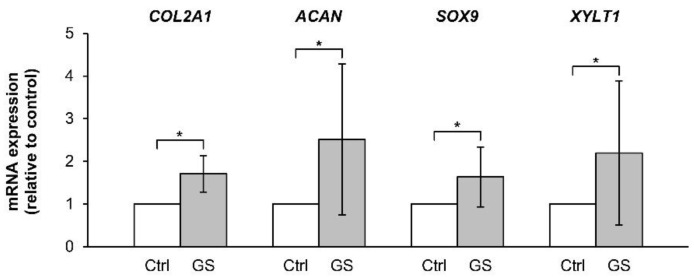
Effects of glucosamine sulfate on the expression of cartilaginous matrix anabolic genes in cytokine-free treated porcine pellet culture. The pellets were cultured for 7 days with glucosamine sulfate (GS; 125 μg/mL). The untreated pellets were left as a control (Ctrl). The data were statistically analyzed using Mann–Whitney *U* test. Data are expressed as mean ± SD of three independent experiments. * = *p* < 0.05.

We further investigated the restored ability of chondrocytes to express anabolic genes in the pellet culture, which was pretreated with the combined cytokines for 3 days prior to culturing in a cytokine-free media with or without glucosamine sulfate for 7 and 14 days (representative diagram of pellet treatment depicted in Figure 14). The expressions of the anabolic genes COL2A1, ACAN, SOX9, and XYLT1 were strongly suppressed by the continuous combined cytokine treatment (Figure 8). When combined cytokine-pretreated pellets were cultured in the cytokine-free media, the expression levels of these anabolic genes were significantly increased to similar levels as that observed in the untreated control group in 1 week of the removal of the combined cytokine stimulation. Under these conditions, the addition of glucosamine sulfate resulted in a further increase in the gene expression levels of the anabolic genes. However, only the expression of XYLT1 gene was found to be statistically significant in the first week of treatment. The expression of these genes decreased when the treatments were extended to 2 weeks. In the absence of cytokine pretreatment, glucosamine sulfate exerted synthetic stimulating effects on the upregulation of the cartilaginous matrix anabolic markers, including COL2A1, ACAN, SOX9, and XYLT1 genes (Figure 9).

**Figure 10:**
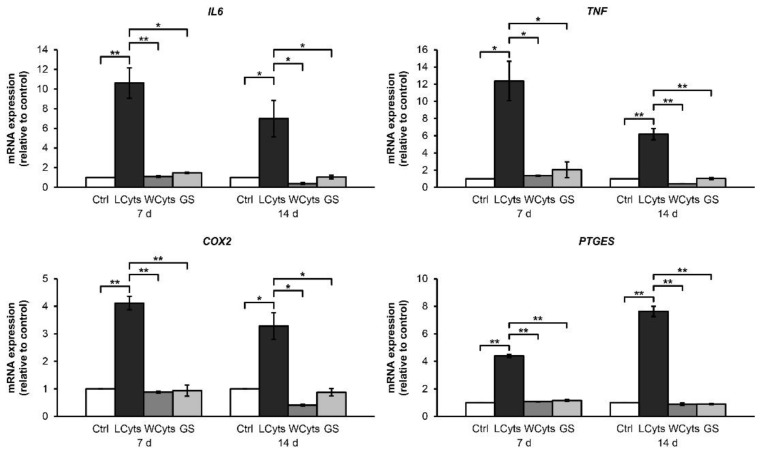
The cytokine-withdrawal effects after pretreatment with the IL-1β and IL-17A combination on the mRNA expression of proinflammatory cytokines and inflammatory mediators in porcine pellet culture. The pellets were pretreated by the combined cytokines (0.25 ng/mL of IL-1β in combination with 0.5 ng/mL of IL-17A) for 3 days, and then cultured for 7 and 14 days by non-cytokine treatments (Wcyts) with or without glucosamine sulfate (GS; 125 μg/mL). The untreated pellets were left as a control (Ctrl). Long-term treatments were continuously cultured until 7 and 14 days (LCyts). Data are expressed as mean ± SD of two independent experiments. * = *p* < 0.05 and ** = *p* < 0.001.

Furthermore, the gene expression levels of proinflammatory cytokines and inflammatory mediators were also investigated (Figure 10). Continuous combined cytokine treatment for 7 and 14 days significantly induced the expressions of IL6, TNF, COX2, and PTGES genes. These effects were reversed when the combined cytokine-pretreated pellets were cultured in the cytokine-free media. Under these conditions, glucosamine sulfate did not alter the expression of any indicated gene.

**Figure 11:**
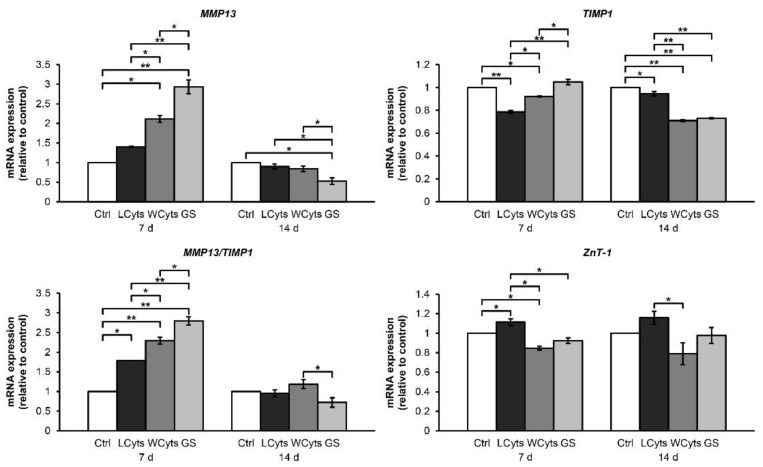
The cytokine-withdrawal effects after pretreatment with the IL-1β and IL-17A combination on the expression of genes associated with cartilaginous matrix-degrading enzymes in porcine pellet culture. The pellets were pretreated by the combined cytokines (0.25 ng/mL of IL-1β in combination with 0.5 ng/mL of IL-17A) for 3 days, and then cultured for 7 and 14 days by non-cytokine treatments (Wcyts) with or without glucosamine sulfate (GS; 125 μg/mL). The untreated pellets were left as a control (Ctrl). Long-term treatments were continuously cultured until 7 and 14 days (LCyts). Data are expressed as mean ± SD of two independent experiments. * = *p* < 0.05 and ** = *p* < 0.001.

Figure 11 illustrates a small increase in the expression levels of MMP13, MMP13/TIMP1, and ZnT-1 when the pellets were treated with combined cytokine treatment for 1 week, whereas the mRNA levels of TIMP1 were lower than those in the untreated control group at 7 and 14 days. These effects were enhanced when the combined cytokine-pretreated pellets were cultured in the cytokine-free media in the presence or absence of glucosamine sulfate at 7 days. These effects were then decreased to basal levels at 14 days. Meanwhile, the expression of TIMP1 and ZnT-1 genes in the cytokine-withdrawal conditions were not upregulated compared with those in the untreated control group.

**Figure 12:**
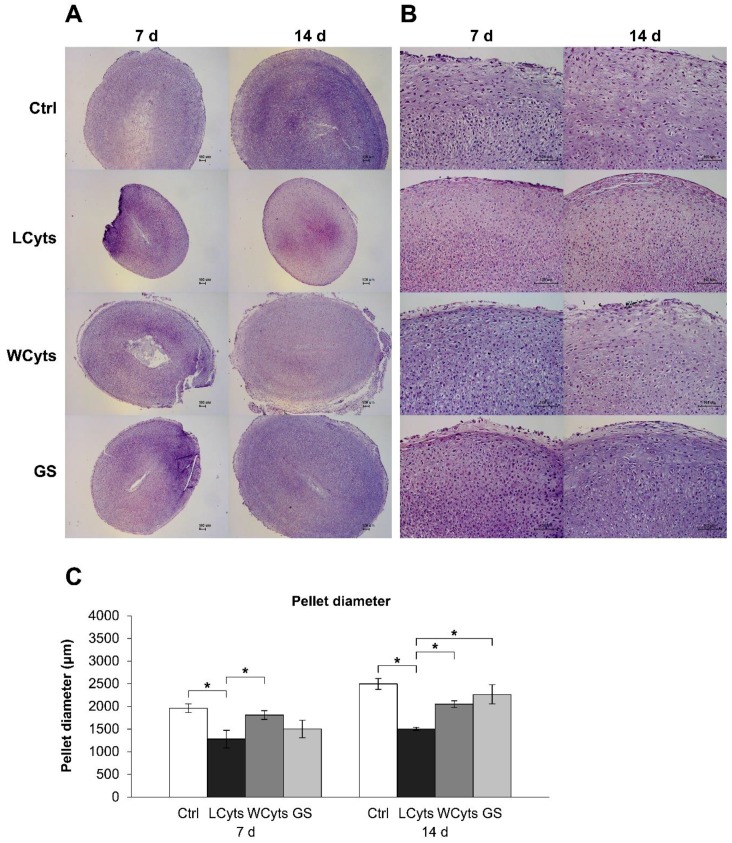
The cytokine-withdrawal effects after pretreatment with the combined IL-1β and IL-17A on the morphology and growth of chondrocytes in porcine pellet culture. The pellets were pretreated by the combined cytokines (0.25 ng/mL of IL-1β in combination with 0.5 ng/mL of IL-17A) for 3 days, and then cultured for 7 and 14 days by non-cytokine treatments (Wcyts) with or without glucosamine sulfate (GS; 125 μg/mL). The untreated pellets were left as a control (Ctrl). Long-term treatments with the combined cytokines were continuously cultured until the end of experiments (LCyts). The whole pellets were stained with H&E and digitized at 5× (**A**) and 20× (**B**) magnifications. The diameters of the pellets were measured along their shortest axis on the 5× magnification images (**C**). Data are expressed as mean ± SD of two independent experiments. * = *p* < 0.05.

**Figure 13:**
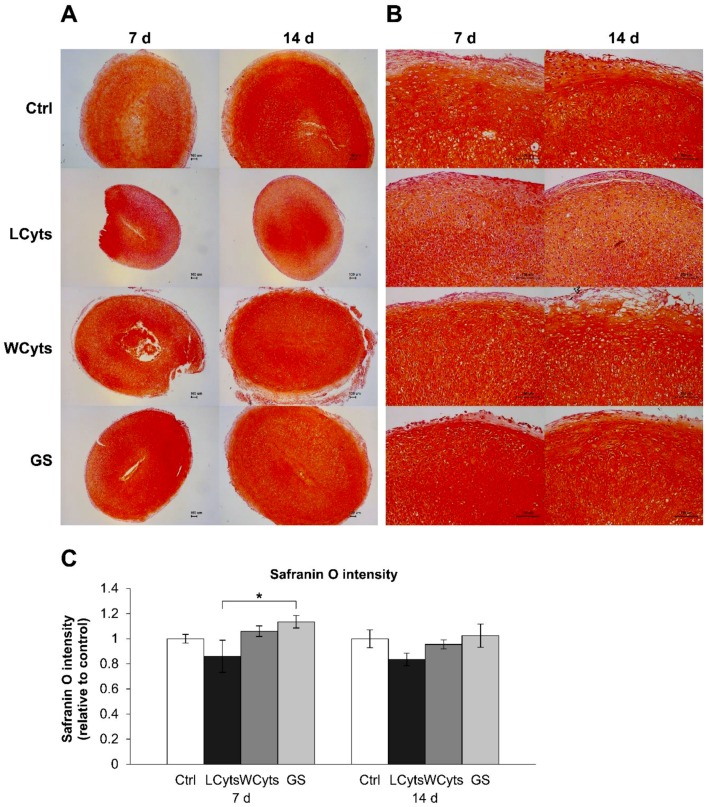
The cytokine-withdrawal effects after pretreatment with the IL-1β and IL-17A combination on the synthesis of proteoglycans in porcine pellet culture. The pellets were pretreated by the combined cytokines (0.25 ng/mL of IL-1β in the combination with 0.5 ng/mL of IL-17A) for 3 days, and then cultured for 7 and 14 days by non-cytokine treatments (Wcyts) with or without glucosamine sulfate (GS; 125 μg/mL). The untreated pellets were left as a control (Ctrl). Long-term treatments with the combined cytokines were continuously cultured until the end of experiments (LCyts). The whole pellets were stained with safranin O dye and digitized at 5× (**A**) and 20× (**B**) magnifications. The intensities of safranin O were calculated on 5x magnification safranin O-stained slides (**C**). Data are expressed as mean ± SD of two independent experiments. * = *p* < 0.05.

To evaluate the changes in chondrocytes in the pellets affected by the combined cytokine treatment, the pellets were stained with H&E for cell morphology assessment. The cytokine treatment groups on 7 and 14 days displayed the smallest pellet size compared with the other groups (Figure 12A). When the cytokines were removed after pretreatment for 3 days and pellets were cultured in the cytokine-free media for 7 and 14 days, the pellet diameters expanded to a size similar to that observed in the untreated control group (Figure 12C). Under these conditions, glucosamine sulfate significantly enhanced the pellet growth during the following 2 weeks of culture. High-magnification H&E-stained sections showed that the chondrocytes had different shape patterns and were rearranged into separate layers (Figure 12B). Flattened cells were observed along the surface of the pellet, round cells were distributed in the middle layers, and cells in a column formation were aligned in the deeper layers of the pellet. Long-term cytokine treatments revealed small-sized cells with a close-packed arrangement. These cells were lost when the cytokine treatment was ceased, regardless of the presence of glucosamine sulfate. Furthermore, the accumulation of proteoglycans in the pellets, indicated by the intensity of staining with safranin O on low-magnification sections, was slightly decreased in the combined cytokine treatment group (Figure 13A,C). Removal of cytokine stimulation caused an increase in the proteoglycan accumulation, which was significantly increased by glucosamine sulfate in comparison with the combined cytokine treatment group at 7 days. High-magnification assessment (Figure 13B) revealed that the pellets treated with the combined cytokine treatment showed a slightly pale intensity of staining for safranin O on their surface area at 7 days, and this staining was expanded to the middle layers of the pellet at 14 days, whereas the other groups showed large accumulations of proteoglycans (observed as a deep intensity of staining for safranin O), which were homogeneously distributed throughout the pellets.

## 3. Discussion

Proinflammatory cytokines play significant roles in the pathogenesis of inflammatory arthritis and escalate the severity of joint inflammation. Studies have shown that the combination of IL-1β and IL-17A has a synergistic effect on joint destruction [9,14]. However, the underlying mechanisms of the action of the combination of IL-1β and IL-17A need to be elucidated. Our study, using in vitro 3D models, demonstrated that the catabolic effects of IL-1β on cartilage explant degradation were enhanced when combined with IL-17A. The cytokine combination was found to effectively suppress the expression of the anabolic genes, including COL2A1, ACAN, SOX9, and XYLT1. These suppressive effects were lost when treatment with the cytokine combination was removed. The catabolic factors associated with inflammatory arthritis, such as proinflammatory cytokines, proinflammatory mediators, and the key enzyme MMP-13, were found to be dramatically increased by the IL-1β and IL-17A combination.

In arthritic joints, the assembly of ECM in the cartilage is disrupted, and S-GAGs are easily dissociated from the collagen fibrils [15]. In the porcine explant model, the increase in S-GAGs released into the culture media accompanied with the reduction of UA and proteoglycan content in the explants indicated the progress of cartilage degradation [16]. Our study clearly revealed that treatment with the cytokine combination, IL-1β and IL-17A, was more effective in activating cartilage degradation than treatment with each proinflammatory cytokine alone. Our findings showed that the cytokine combination could trigger the process of cartilage degradation as early as within 6 h and the degradation reached significant levels at 24 h, as determined by the significant release of S-GAGs together with the high expression of MMP13. The S-GAGs levels increased continuously to significant levels up to 7–14 days and gradually declined later. In comparison with the first 3 h of cartilage culture, the levels of S-GAGs release in the untreated control group gradually increased with time possibly because of the sensitivity of cartilage explants to environmental changes after its removal from the closed system of animal joints, followed by a spontaneous degradation in the culture system. Therefore, the release of cartilaginous matrix molecules, particularly S-GAGs, was observed throughout the study. These findings are beneficial for cartilage research, especially for the identification of the in vitro chondroprotective properties of some agents by applying the cytokine pretreatments for at least 6 h prior to cotreatment with the test compound for at least 1 week. However, some agents, such as diacerein, which counteracted the effect of the combined cytokines in the late stages of the experiments, should be analyzed with respect to their levels of accumulation. The findings of our study demonstrated that diacerein, a slow-acting anti-inflammatory drug, exhibited chondroprotective properties against the effects of the combined cytokines, as reported previously [17].

MMP-13 is one of the key enzymes involved in cartilage and bone erosion. MMP13 gene expression is inducible and enhanced by the activation of proinflammatory cytokines [18]. The coexistence of the high expression of MMP13 gene together with the significant release of S-GAGs in the combined cytokine-treated cartilage explants indicated the initial period of cartilage degradation. MMP-13 levels in the cartilage explant culture media were still high until the end of the long-term treatments with the combined cytokines, while the levels in the untreated control were found to decrease. This may suggest multiple levels of regulation of this enzyme from post-transcriptional regulation [19]. The weekly assessment of MMP13 expression in the explant culture model should be further investigated together with other matrix-degrading molecules, which are involved in cartilage degeneration diseases, including MMP-1and MMP-3.

Further evidence of cartilage degradation that was accelerated by the combined cytokines was provided by the histopathological examination of the cartilage explants section with H&E and safranin O staining. The H&E-stained slides showed normal morphology of the chondrocytes in all the groups. Nevertheless, the nucleated cell count on the H&E-stained slides was valuable for viable cell investigation. The combined cytokine-treated group showed the lowest number of surviving chondrocytes. This may be because the cells may have undergone programmed cell death, which is an apoptotic induction by the proinflammatory cytokines on the chondrocytes [20]. Nevertheless, the levels of LDH release in all the treated groups did not differ from those in the untreated control group, suggesting a limitation of the LDH assay in evaluating the viability of embedded chondrocytes in cartilage explants. The integrity of the proteoglycans remaining in the treated cartilage explants was observed by safranin O staining. Safranin O dye binds to the entire proteoglycans in the cartilage tissue indicated the remaining ECM content by the end of experiment [21]. Treatment with the cytokine combination was obviously the reason for ECM loss, which could be observed by the pale intensity of safranin O and depleted contents of UA and collagen. The ECM composition in the porcine cartilage explants was preserved by diacerein, which was effective as a positive control.

Regarding the cartilage explant model, which was adopted for studying cytokine-induced cartilage degradation, the porcine cartilage obtained from a local market was convenient and affordable. The genetic similarities between pigs and humans make them a potential animal model for studying human diseases [22]. The cartilage explant culture provides intact cartilage, which represents the actual chondrocyte metabolism and mechanisms in the pathological progression of cartilage degeneration. However, the turnover and the regeneration of the chondrocytes were slow and difficult to observe. Thus, studies on the cartilaginous matrix biosynthesis were rather limited when using the cartilage explant model. Previously, a radioactive substance was used for evaluating the proteoglycan synthesis in a bovine cartilage explant [23]. We attempted to use a non-radioactive technique for investigating the cartilaginous ECM synthesis at the molecular level using the pellet culture model. This model has been used for studying the molecular mechanisms of cartilaginous matrix turnover because of the steady chondrogenic phenotype [24,25]. Several experiments have been performed with pellet cultures from bovine, equine, and human chondrocytes in physiological and pathological conditions, in which IL-1β was used for the inflammatory induction [26,27,28].

Our study unveiled the different expression patterns of genes associated with cartilage matrix synthesis in parallel with the expression of the catabolic factors in porcine pellet cultures under long-term treatment with IL-1β and IL-17A in combination. Because of the direct exposure of the cytokines to chondrocytes in the pellet model, the eightfold lower concentration of the combined cytokines compared to the explant culture model was appropriate for use in long-term studies of the expression of anabolic and catabolic genes. However, long-term treatment with the combined cytokines in pellet culture yielded inaccurate results because the pellet size in the combined cytokines group certainly differed from that of the untreated control (Figure S3). The present study shows that treatment with the combined cytokines in porcine pellet culture for 3–14 days is sufficient for studying both the catabolic and anabolic gene expression levels.

The cartilage-specific anabolic genes defined as the chondrogenic markers are COL2A1, ACAN, SOX9, and XYLT1 genes, which encode type II collagen, aggrecan core protein, transcription factor SOX-9, and xylosyltransferase I enzyme, respectively [29,30]. These genes regulate chondrogenesis and their expression levels are found to increase during cartilage development. A previous study showed that pretreatment of IL-1β in human articular chondrocyte pellets suppressed the expression of the ACAN gene and upregulated the expression of the catabolic genes [31]. Nevertheless, no report exists on the induction of the cytokine combination on the anabolic mechanisms, and the changes after cytokine withdrawal are still debatable. Our study shows the combinatorial effect of IL-1β and IL-17A on the expression of the genes associated with cartilage regeneration under stress conditions, and the ability of the chondrocytes to restore the gene expression when the cytokine combination was withdrawn. As long as the combined cytokines remained in the pellet culture, the expression of the cartilage-specific anabolic genes reduced continuously, especially that of the COL2A1 gene. After pretreatment with the combined cytokines for 3 days followed by removal of the cytokines, the expression of the anabolic genes was restored to nearly that of the untreated control at 7 days. These results suggest that the affected chondrocytes are able to regenerate the cartilage matrix after the inflammatory process is resolved by anti-inflammatory drugs, which are normally prescribed for RA. Accordingly, in-depth investigation of these matrix regenerations is required.

Glucosamine sulfate, which reportedly activates the anabolic genes of chondrocytes [32], showed a weak trend in restoring the expression of the anabolic genes, except for XYLT1. Interestingly, under culture conditions without cytokines, glucosamine sulfate significantly upregulated the cartilage-specific anabolic genes in a week, as previously reported [24]. We postulated that there are some changes in the cytokine-treated chondrocytes, leading to the lower responsiveness of the cells in response to activation by glucosamine sulfate. Our results also suggested that the duration for evaluating the restorative effects on cartilaginous matrix biosynthesis by the present pellets culture model should be achieved within a week after pretreatment with the combined cytokines.

In the long-term treatment of the pellets with the cytokine combination, although a significant upregulation of mRNA expression of arthritis-related proinflammatory cytokines, IL-6 and TNF-α, together with the mediator, COX2, was detected within 24 h, a striking increase in the expression of these genes was demonstrated after 2 weeks. TNF-α and IL-6 play a pivotal role in the pathophysiology of RA [33,34]. PGE2, a proinflammatory mediator encoded by the COX2 gene, is one of the key mediators that contributes to the pathogenesis of arthritis such as inflammation and angiogenesis [35,36]. Since PGE2 induces the expression of IL-6 and IL-17 [37,38], the massive expansion of these proinflammatory cytokines when the pellets are continuously exposed to the combined cytokines may suggest a network amplification of the proinflammatory cytokines and mediators during chronic inflammatory arthritis, especially RA.

Unlike the anabolic genes, which were restored after the removal of the cytokine combination treatment, the high expression levels of the proinflammatory cytokines were completely lost. These findings suggested that the amplification chain in the production of proinflammatory cytokines could be weakened by reducing the amount of the key proinflammatory cytokines and by using anti-inflammatory agents that counteract the effects and production of the crucial proinflammatory cytokines, IL-1β and TNF-α, in OA and RA, respectively [39,40]. The inhibitor cocktail of proinflammatory cytokines seemed to be effective; however, the complete inhibition of cytokines is a matter of concern, because these cytokines are essential for the general immune responses [41,42]. This study demonstrates the ability of IL-17A to be an enhancer of IL-1β in inflammatory arthritis. Therefore, the balance between anti-inflammation and inflammation targeting the suppression of the inflammatory enhancers like IL-17A should be considered, rather than the complete inhibition of the key cytokines.

One of the pivotal phenomena occurring in arthritis is cartilage degradation. Both OA and RA progression end with cartilage damage caused by matrix-degrading enzymes [43]. During joint inflammation, the inducible MMP13 gene and zinc ion transporter in porcine chondrocytes, ZnT-1, are stimulated by various proinflammatory cytokines and inflammatory mediators. The catalytic domain of MMP-13 requires Zn^2+^ as a cofactor for substrate cleavage [44]. Since TIMP1 is an endogenous tissue inhibitor of MMP-13, the ratio of MMP13/TIMP1 genes implies the level of cartilage degradation [8]. Consistently with the cartilage explant model, the pellet culture model showed a high mRNA expression of MMP13 and the ratio of MMP13/TIMP1 for only 1–3 days after treatment with the combined cytokines and continuously declined until 28 days. The levels of TIMP1 and ZnT-1 genes increased in a similar cyclical pattern. Unexpectedly, after pretreatment with the cytokine combination and followed by cultivation in culture media without the cytokines for 7 days, the expression of MMP13 and the ratio of MMP13/TIMP genes were persistently upregulated beyond that of the groups in which the pellets were continuously exposed to the combined cytokines. In the pellet culture model, the porcine chondrocytes assembled into a rounded structure, whereby the centric cells in the pellets became short of sufficient nutrients and finally died. The death of chondrocytes triggers inflammation and the production of proteolytic enzymes [20]. Although the gene expression of the proinflammatory cytokines and inflammatory mediators were entirely lost on cytokine withdrawal, we postulated that the MMP13 gene may be stimulated by the chondrocytic death pathways. Nevertheless, the treatments that extended to 2 weeks failed to assess MMP13 gene expression. Thus, the effect of matrix-degrading enzymes in the pellet culture should be studied in the early phase of inflammation in order to elucidate the accurate metabolism of chondrocytes.

Our study showed that the morphology and arrangement of the chondrocytes in the pellets were similar to those in the normal cartilage tissue, which is divided into three zones in different arrangements [45]. Within the 3D pellet culture, the accumulation of proteoglycans, which indicates the synthesis of proteoglycans, was observed. This finding suggests that the chondrogenic property of the chondrocytes is well preserved in this system. The cytokine combination clearly affected the chondrocyte morphology, arrangement, and metabolism as it resulted in the reduction of the pellet size and the accumulation of proteoglycans. The extracellular spaces were found to decrease together with the increased accumulation of the small cells. This may be due to the disturbance in the chondrogenic properties of the cells by the cytokine combination, thereby leading to an alteration in the biomechanical environment of the chondrocytes. This was supported by the regain in the pellet size, chondrocyte arrangement, and proteoglycan accumulation after cytokine withdrawal. Nevertheless, a small reduction in safranin O color intensity was observed for the combined cytokine treatment group compared with the other groups on both 7 and 14 days of cultures. Therefore, evaluation of proteoglycans accumulation (red stain) in the pellet culture model may not be a suitable assay owing to its low sensitivity.

In summary, our study provides additional evidence regarding proinflammatory cytokines, including IL-1β and IL-17A, which are found in the microenvironment of arthritic joints. These cytokines may act as a team to provoke the pathology of inflammatory arthritis by upregulating the expression of proinflammatory cytokines and cartilage-degrading enzymes, while suppressing the expression of the genes associated with cartilage regeneration, thereby leading to cartilage and joint destruction in chronic inflammatory arthritis, especially RA. The withdrawal of this cytokine combination could resolve the consequence of inflammation and restore the capability of the chondrocytes to synthesize the cartilage matrix molecules. Therefore, we propose that reducing the enhancing effects of IL-17A in parallel with reactivating the process of cartilage regeneration may be the gold target for counteracting the pathogenesis of arthritis. In addition, our study shows the 3D models of chondrocyte culture, which were activated by the combination of IL-1β and IL-17A. These systems might be useful and applicable for cartilage research studies.

## 4. Materials and Methods

### 4.1. Chemical Reagents

All cultured reagents were purchased from Gibco (Grand Island, NY, USA). Recombinant human IL-1β and IL-17A were obtained from ProSpec Protein Specialist (Ness-Ziona, Israel).

### 4.2. Porcine Cartilage Explant Culture

The domestic swine feet were obtained from Chiang Mai slaughterhouse, Chiang Mai, Thailand within 6 h post mortem. The feet were disinfected in bleach for 15 min; next, the outer skin was removed. The skinned feet were soaked in 70% ethanol under sterile conditions in a biological safety cabinet. A sterile surgical blade was used for dissecting the cartilage discs from the metacarpophalangeal joints. All cartilage discs were soaked in serum-free DMEM with 1000 U/mL penicillin-streptomycin (Gibco) for 30 min and then incubated in serum-free DMEM in a humidified incubator (37 °C and 5% CO_2_) for 24 h. After the sterility check, three cartilage discs were randomly grouped per well of a 24-well culture plate to totally weigh 30–35 mg (Figure S1) and cultured with serum-free DMEM for 24 h. The explants were treated with IL-1β or/and IL-17A. Diacerein (Artrodar), an anti-arthritis drug, was used as the positive control at 50 μM. Culture media were collected and replaced with the same treatments every 7 days. The cartilage discs were harvested at the end of the treatments.

### 4.3. Evaluation of Sulfated Glycosaminoglycans Levels

A massive release of sulfated glycosaminoglycans (S-GAGs) represents cartilage matrix degradation in a pathological condition. Measuring the levels of S-GAGs using dimethylmethylene blue (DMMB) assay in culture media was performed as previously described [46]. Chondroitin sulfate C (Sigma-Aldrich, St. Louis, MO, USA) was used as the standard for the calculation of S-GAGs concentrations.

### 4.4. Uronic Acid and Collagen Assays

The contents of proteoglycans and collagen in the cartilage indicate the integrity of the cartilage tissue. Loss of these macromolecules is usually referred to as severe cartilage degradation. Dried and treated cartilage discs were digested with 10 U/mL of papain (Sigma-Aldrich) at 60 °C for 24 h. The digested samples were diluted 120-fold with deionized water and subjected to the carbazole reaction for uronic acid assay as previously described [47]. Glucuronic acid lactone (Sigma-Aldrich) was used as the standard for the calculation of UA concentrations. The collagen contents of the digested samples were measured using colorimetric hydroxyproline assay [48]. Hydroxyproline (Sigma-Aldrich) was used for standard curve analysis.

### 4.5. Analysis of MMP-13 Levels

The levels of MMP-13 protein in the culture media were determined by ELISA using the MMP-13 CLIA Kit (Elabscience, Houston, TX, USA). The analysis was performed in accordance with the manufacturer’s instructions.

### 4.6. Viability Testing via Lactate Dehydrogenase Quantification

Cartilage viability was determined by the level of cytoplasmic lactate dehydrogenase (LDH) enzyme released in the cartilage explant culture media. The procedure was performed as previously described [49]. Dimethyl sulfoxide (10%, RCI Labscan, Thailand) was used as the positive control.

### 4.7. Isolation of Porcine Articular Chondrocytes

Porcine cartilage discs were finely chopped in a cell culture dish and digested with 2 mg/mL collagenase type II (Worthington Biochemical, NJ, USA) in a humidified incubator (37 °C and 5% CO_2_) for 24 h. The supernatant was centrifuged at 2000 rpm for 5 min and washed with 1X PBS. The cell pellet was resuspended with 5% fetal bovine serum in DMEM and cultured in 75 cm^2^ culture flasks.

### 4.8. Pellet Culture from Porcine Articular Chondrocytes

**Figure 14:**
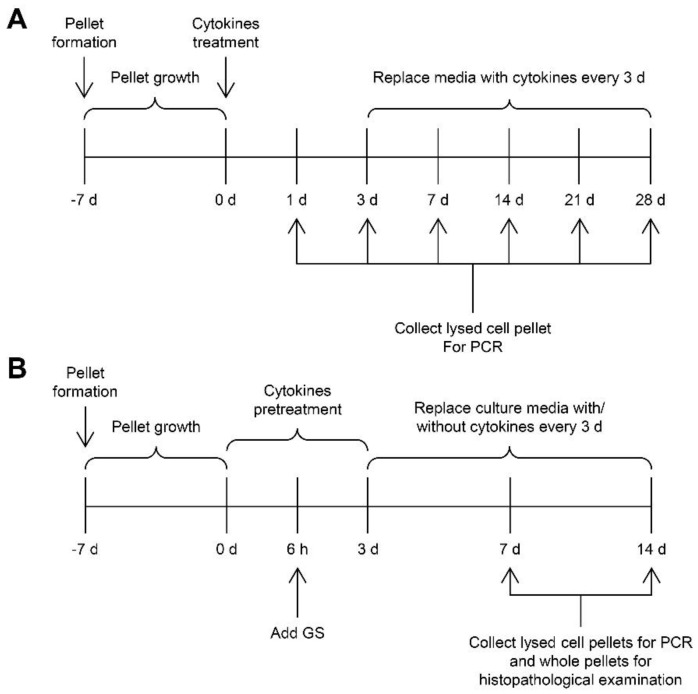
Temporal diagrams of the pellet experiments demonstrate the study of the periodical effects of the combined cytokine treatments on the pellets from primary porcine chondrocytes (**A**), and a study of the recovering effects of cytokine-free treatment and glucosamine sulfate on cytokine-treated pellets (**B**).

Primary porcine chondrocytes at passage 2–3 were trypsinized, washed with 1X PBS, and centrifuged at 2000 rpm for 5 min. Cells were resuspended in a chondrogenic medium (Table S1) and transferred to a 15 mL conical polypropylene centrifuge tube at a concentration of 1 × 10^6^ cells per milliliter of the medium. Tubes were centrifuged at 1500 rpm for 5 min and incubated in a humidified incubator (37 °C and 5% CO_2_) for 1 week. The chondrogenic medium was changed every 3 days. Next, the spherically formed pellets (Figure S2) were treated with a combination of IL-1β and IL-17A for 1–28 days to study the periodical effects, and these treatment media were changed every 3 days. At the designated date, the whole pellets and pellets lysed with a lysis buffer were collected for histopathological examination and gene expression quantification, respectively. After 6 h of cytokine pretreatments, the pellets were cotreated with glucosamine sulfate (Viartril-S, a positive control drug) at 125 μg/mL for 3 days followed by incubation with glucosamine sulfate in cytokine-free media until 7 and 14 days. The diagrams of the pellet experiments are shown below (Figure 14).

### 4.9. Histopathological Examination

The treated cartilage discs or pellets were preserved in 4% (*v/v*) formaldehyde (RCI Labscan) and sent for paraffin embedding, microtome sectioning, and hematoxylin-eosin (H&E) staining at the Department of Pathology, Faculty of Medicine, Chiang Mai University, Thailand. Viable cells (nucleated lacunae) were counted on the H&E-stained slides related to the total cells (nucleated and non-nucleated lacunae). Unstained slides were stained with safranin O dye (Sigma-Aldrich) for investigating the remaining proteoglycans. The safranin O color intensity was determined using the ImageJ software [50].

### 4.10. Quantification of Arthritis Gene Expressions

The treated cartilage discs were ground to fine powder in liquid nitrogen with a mortar and pestle. The ground cartilages or pellets were suspended in a lysis solution for total RNA extraction using the innuPREP RNA Mini Kit (Analytik Jena, Jena, Germany). cDNA was synthesized using the ReverTra Ace qPCR RT master mix (TOYOBO, Osaka, Japan). Specific primers and SensiFast SYBR No-ROX reagent (Bioline, London, UK) were used for real-time PCR on 7500 Fast Real-time PCR system (Applied Biosystems). The porcine primer sequences (Table S2) were designed from the associated GenBank accession numbers using the NCBI Primer-BLAST tool [51]. Data were calculated using the 2^−ΔΔCt^ method and normalized with the GAPDH reference gene.

### 4.11. Statistical Analysis

Data were expressed as mean ± standard deviation of two or three independent experiments. The significance was analyzed using Mann–Whitney *U* test and one-way analysis of variance followed by post hoc Tukey multiple comparison test when appropriated by the IBM SPSS statistics software version 22 (IBM Corp., Armonk, N.Y., USA). *p*-values < 0.05 (#, *) and 0.001 (##, **) were considered statistically significant.

## Supplementary Materials

The following are available online, Figure S1: The porcine cartilage explant in 24-well culture plate, Figure S2: The porcine pellet culture in chondrogenic medium at 7 days, Figure S3: Pellet size in long-term treatment, Figure S4: Dose-response and time-course effects of the IL-1β and IL-17A combination on the expression of cartilaginous matrix anabolic genes in porcine pellet culture, Figure S5: Dose-response and time-course effects of the IL-1β and IL-17A combination on the mRNA expression of proinflammatory cytokines and inflammatory mediators in porcine pellet culture, Figure S6: Dose-response and time-course effects of the IL-1β and IL-17A combination on the expression of the genes associated with the cartilaginous matrix-degrading enzymes in porcine pellet culture, Table S1: The compositions of chondrogenic medium, Table S2: The porcine primer sequences.
