# Supplementary material for: Proinflammatory Effects of IL-1β Combined with IL-17A Promoted Cartilage Degradation and Suppressed Genes Associated with Cartilage Matrix Synthesis In Vitro
Source: Molecules. 2019 Oct 13;24(20):3682. doi: 10.3390/molecules24203682 (PMC6833041; doi:10.3390/molecules24203682)
Supplement: Supplementary file 1 [file molecules-24-03682-s001.pdf]

Supplementary materials

Table S1. The compositions of chondrogenic medium.

Ingredients	Final concentration
Fetal bovine serum (Thermo Fisher Scientific)	10%
Insulin-Transferrin-Selenium, 100x (Sigma-Aldrich)	1X
Ascorbic acid (Sigma-Aldrich)	25 μg/mL
Dexamethasone (Sigma-Aldrich)	0.1 μM

Table S2. The porcine primer sequences.

Genes	Accession number	Primer sequences
GAPDH	NM_001206359.1	F: 5′-CAGGTTGTGTCCTGTGACTT-3′<br>R: 5′-CCTGTTGCTGTAGCCAAATTCAT-3′
COL2A1	XM_001925959.6	F: 5′-GAAGACCTGAAAGACTGCCTC-3′<br>R: 5′-TTTGTCACCACGATCACCTCT-3′
ACAN	NM_001164652.1	F: 5′-CACCATCCCCTGCTACTTCA-3′<br>R: 5′-TAGGCACTGTTGACACGCAC-3′
SOX9	NM_213843.1	F: 5′-AGGAAGTCGGTGAAGAACGG-3′<br>R: 5′-GATGGCGTTGGGAGAGATGT-3′
XYLT1	XM_021086555.1	F: 5′-ATCCCGTCAACATCATCGCA-3′<br>R: 5′-AAGGTGGCTTGTAGTGGGTG-3′
IL6	NM_001252429.1	F: 5′-TGTGAAAACAGCAAGGAGGT-3′<br>R: 5′-AAGCATCCGTCTTTTTCTGCC-3′
TNF	NM_214022.1	F: 5′-ATCTACCTGGGAGGGGTCTT-3′<br>R: 5′-TAGTCGGGCAGGTTGATCTC-3′
COX2	NM_214321.1	F: 5′-ATCCTCCGACAGCCAAAGAC-3′<br>R: 5′-AAAGCGGAGGTGTTCAGGAG-3′
PTGES	NM_001038631.1	F: 5′-ACATGGAGACCATTTACCCCTT-3′<br>R: 5′-CCCAGGAAGAAGATCAGGAAGT-3′
MMP13	XM_003129808.4	F: 5′-TCCTGAAGAAAACGGCAGCAA-3′<br>R: 5′-TCCAGTCACCTCTAAGCCGAA-3′
TIMP1	NM_213857.1	F: 5′-TTCATCTACACCCCCGCCAT-3′<br>R: 5′-TATGAGAAACTCCTGGCTGCG-3′
ZnT-1	NM_001139470.1	F: 5′-GTGTTCCTGACCCCTGCAAA-3′<br>R: 5′-AGCAAGGACCAGCCTCATAAA-3′

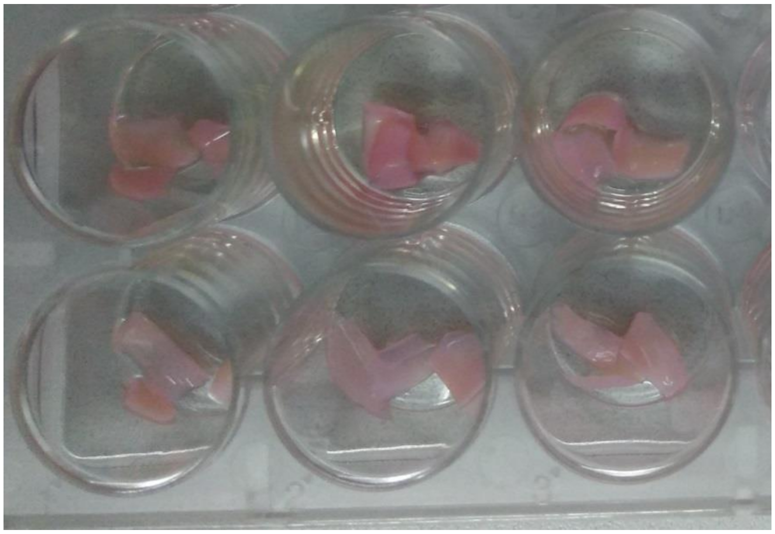

Figure S1. The porcine cartilage explant in 24-well culture plate. Three cartilage discs were randomly grouped in one well with 30-35 mg total weight.

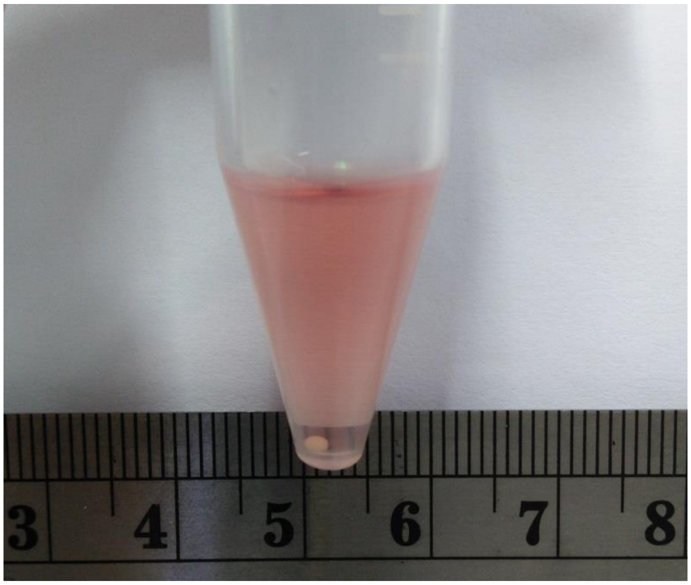

Figure S2. The porcine pellet culture in chondrogenic medium at 7 days. The pellet was formed and maintained in a growth period for up to 7 days in chondrogenic medium, which was changed every 3 days.

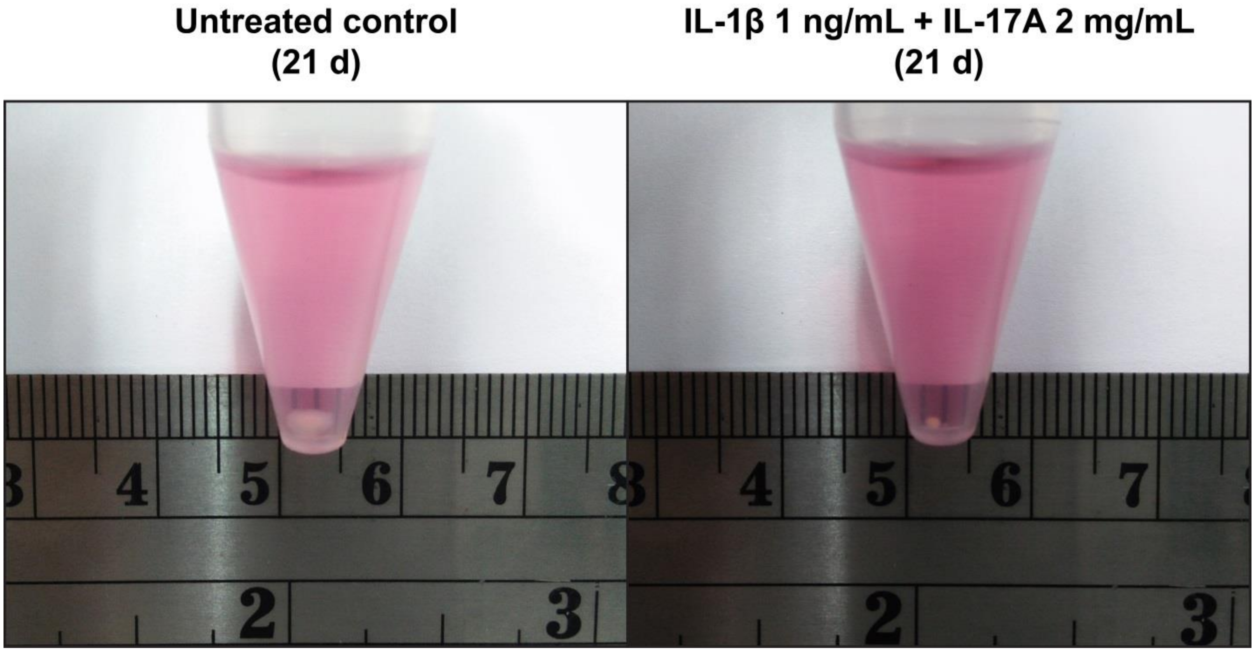

Figure S3. Pellet size in long-term treatment. The pellets were cultured for up to 21 days under conditions in which they were stimulated by the combined cytokines (1 ng/mL of IL-1β in combination with 2 ng/mL of IL-17A). The untreated pellets were left as a control.

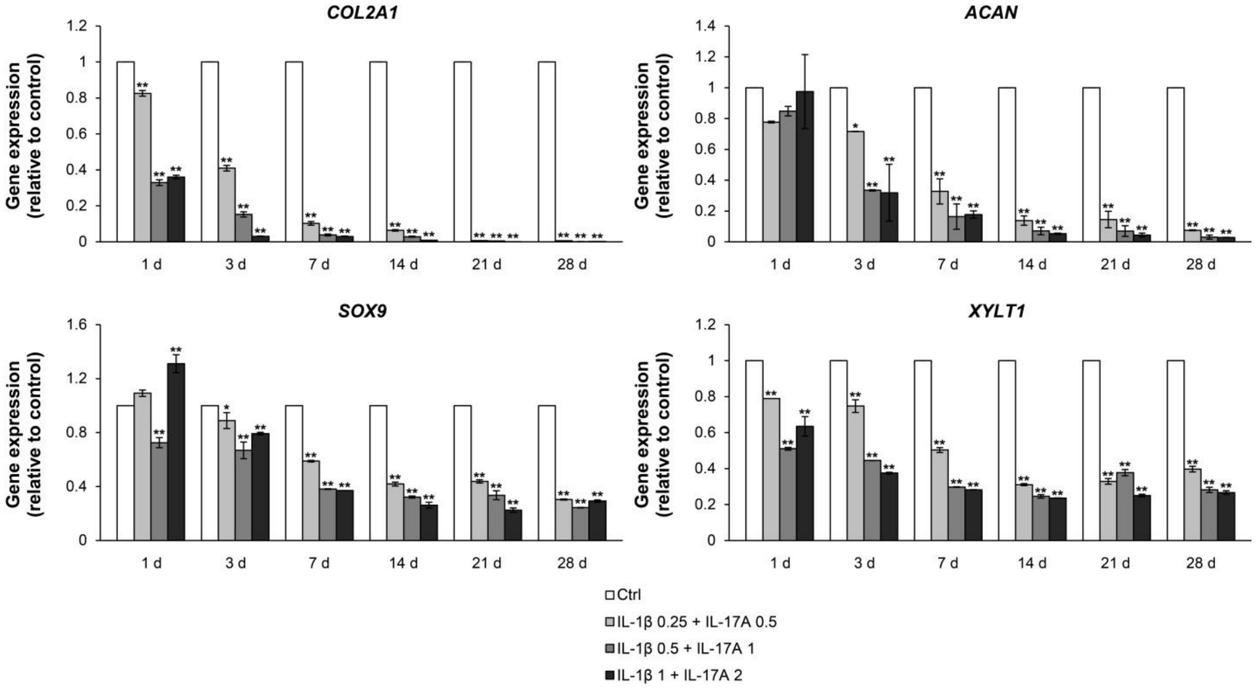

Figure S4. Dose-response and time-course effects of the IL-1β and IL-17A combination on the expression of cartilaginous matrix anabolic genes in porcine pellet culture. The pellets were cultured for up to 28 days under conditions in which they were stimulated by two-fold serial dilutions of the combined cytokines, 2 ng/mL of IL-1β in combination with 4 ng/mL of IL-17A. The untreated pellets were left as a control (Ctrl). The mRNA expressions of the pellets were analyzed by real-time qRT-PCR at indicated times. * = $p < 0.05$ and ** = $p < 0.001$.

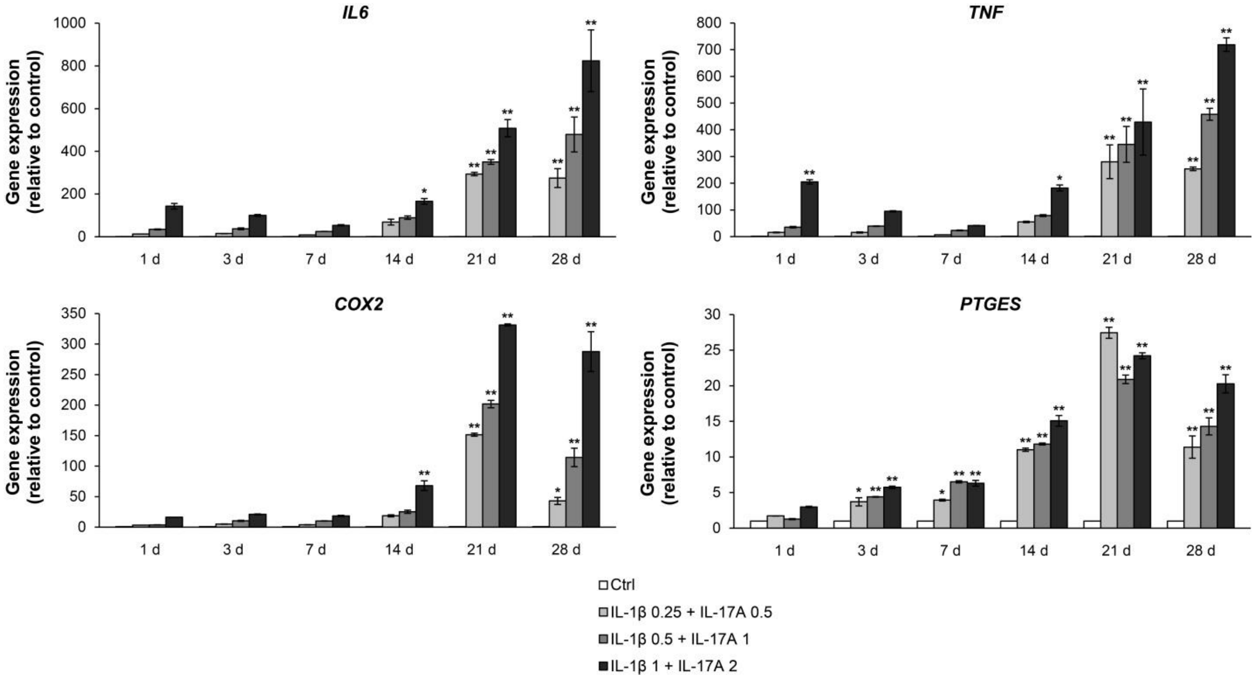

Figure S5. Dose-response and time-course effects of the IL-1β and IL-17A combination on the mRNA expression of proinflammatory cytokines and inflammatory mediators in porcine pellet culture. The pellets were cultured for up to 28 days under conditions in which they were stimulated by two-fold serial dilutions of the combined cytokines, 2 ng/mL of IL-1β in combination with 4 ng/mL of IL-17A. The untreated pellets were left as a control (Ctrl). The mRNA expressions of the pellets were analyzed by real-time qRT-PCR at indicated times. * = $p < 0.05$ and ** = $p < 0.001$.

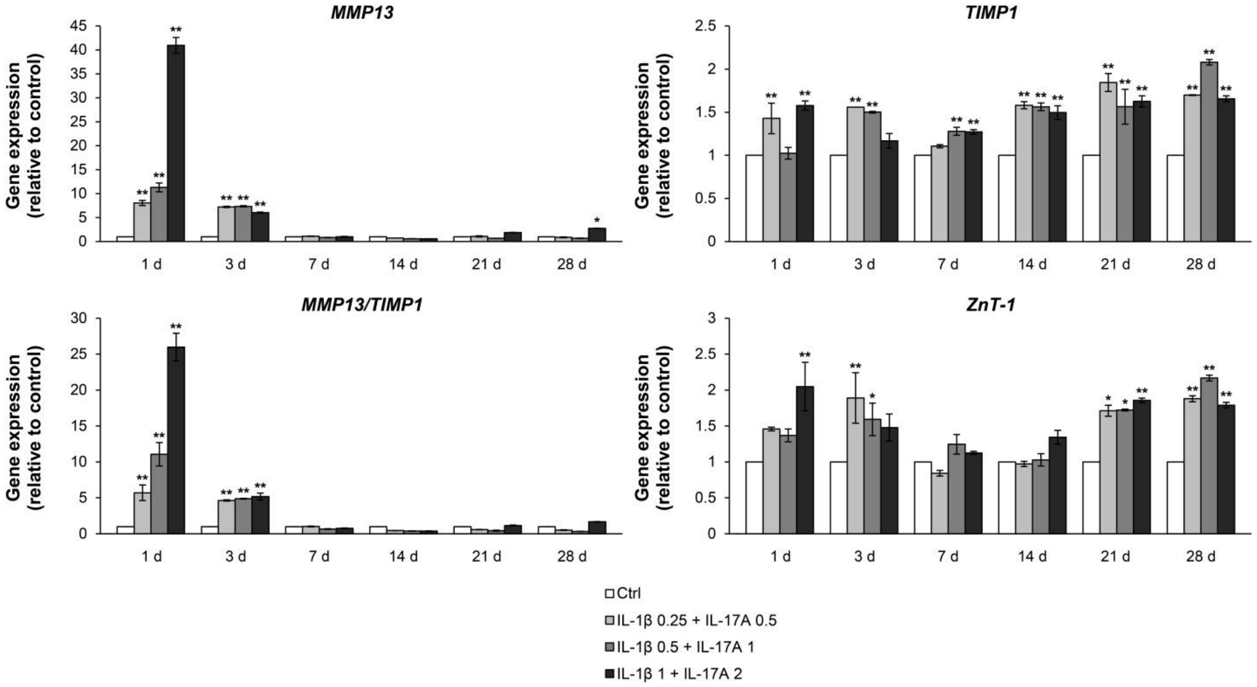

Figure S6. Dose-response and time-course effects of the IL-1β and IL-17A combination on the expression of the genes associated with the cartilaginous matrix-degrading enzymes in porcine pellet culture. The pellets were cultured for up to 28 days under conditions in which they were stimulated by two-fold serial dilutions of the combined cytokines, 2 ng/mL of IL-1β in combination with 4 ng/mL of IL-17A. The untreated pellets were left as a control (Ctrl). The mRNA expressions of the pellets were analyzed by real-time qRT-PCR at indicated times. * = $p < 0.05$ and ** = $p < 0.001$.
